# GDF15 antagonism limits severe heart failure and prevents cardiac cachexia

**DOI:** 10.1093/cvr/cvae214

**Published:** 2024-09-23

**Authors:** Minoru Takaoka, John A Tadross, Ali B A K Al-Hadithi, Xiaohui Zhao, Rocío Villena-Gutiérrez, Jasper Tromp, Shazia Absar, Marcus Au, James Harrison, Anthony P Coll, Stefan J Marciniak, Debra Rimmington, Eduardo Oliver, Borja Ibáñez, Adriaan A Voors, Stephen O’Rahilly, Ziad Mallat, Jane C Goodall

**Affiliations:** Department of Medicine, Victor Phillip Dahdaleh Heart and Lung Research Institute, University of Cambridge, Cambridge CB2 0QQ, UK; Metabolic Diseases Unit, Wellcome-MRC Institute of Metabolic Science and Medical Research Council, University of Cambridge, Cambridge, UK; Department of Histopathology, East Midlands & East of England Genomic Laboratory, Cambridge, UK; Department of Medicine, Victor Phillip Dahdaleh Heart and Lung Research Institute, University of Cambridge, Cambridge CB2 0QQ, UK; Department of Medicine, Victor Phillip Dahdaleh Heart and Lung Research Institute, University of Cambridge, Cambridge CB2 0QQ, UK; Centro Nacional de Investigaciones Cardiovasculares (CNIC), Madrid, Spain; University of Groningen, University Medical Centre Groningen, Groningen, the Netherlands; Saw Swee Hock School of Public Health, National University of Singapore & the National University Health System, Singapore; Department of Medicine, Victor Phillip Dahdaleh Heart and Lung Research Institute, University of Cambridge, Cambridge CB2 0QQ, UK; Department of Medicine, Victor Phillip Dahdaleh Heart and Lung Research Institute, University of Cambridge, Cambridge CB2 0QQ, UK; Department of Medicine, Victor Phillip Dahdaleh Heart and Lung Research Institute, University of Cambridge, Cambridge CB2 0QQ, UK; Metabolic Diseases Unit, Wellcome-MRC Institute of Metabolic Science and Medical Research Council, University of Cambridge, Cambridge, UK; Cambridge Institute for Medical Research, Cambridge Biomedical Campus, University of Cambridge, Cambridge, UK; Metabolic Diseases Unit, Wellcome-MRC Institute of Metabolic Science and Medical Research Council, University of Cambridge, Cambridge, UK; Centro Nacional de Investigaciones Cardiovasculares (CNIC), Madrid, Spain; Centro de Investigaciones Biomédicas en Red de Enfermedades Cardiovasculares (CIBERCV), Madrid, Spain; Centro de Investigaciones Biologicas Margarita Salas (CIB-CSIC), Madrid, Spain; Centro Nacional de Investigaciones Cardiovasculares (CNIC), Madrid, Spain; Centro de Investigaciones Biomédicas en Red de Enfermedades Cardiovasculares (CIBERCV), Madrid, Spain; IIS-Hospital Fundacion Jimenez Diaz, Madrid, Spain; University of Groningen, University Medical Centre Groningen, Groningen, the Netherlands; Metabolic Diseases Unit, Wellcome-MRC Institute of Metabolic Science and Medical Research Council, University of Cambridge, Cambridge, UK; Department of Medicine, Victor Phillip Dahdaleh Heart and Lung Research Institute, University of Cambridge, Cambridge CB2 0QQ, UK; Paris Cardiovascular Research Center, Université Paris Cité, INSERM UMRS 970, Paris, France; Department of Medicine, Victor Phillip Dahdaleh Heart and Lung Research Institute, University of Cambridge, Cambridge CB2 0QQ, UK

**Keywords:** Heart failure, GDF15, PPP1R15A, Cachexia, Integrated stress response

## Abstract

**Aims:**

Heart failure and associated cachexia is an unresolved and important problem. This study aimed to determine the factors that contribute to cardiac cachexia in a new model of heart failure in mice that lack the integrated stress response (ISR) induced eIF2α phosphatase, PPP1R15A.

**Methods and results:**

Mice were irradiated and reconstituted with bone marrow cells. Mice lacking functional PPP1R15A, exhibited dilated cardiomyopathy and severe weight loss following irradiation, whilst wild-type mice were unaffected. This was associated with increased expression of *Gdf15* in the heart and increased levels of GDF15 in circulation. We provide evidence that the blockade of GDF15 activity prevents cachexia and slows the progression of heart failure. We also show the relevance of GDF15 to lean mass and protein intake in patients with heart failure.

**Conclusion:**

Our data suggest that cardiac stress mediates a GDF15-dependent pathway that drives weight loss and worsens cardiac function. Blockade of GDF15 could constitute a novel therapeutic option to limit cardiac cachexia and improve clinical outcomes in patients with severe systolic heart failure.

**Time of primary review: 28 days**

## 1. Introduction

Heart failure (HF) is a common, complex condition with a poor prognosis and increasing incidence.^1^ Cachexia is associated with chronic heart failure and its occurrence independently predicts increased morbidity and mortality independently of other variables.^2^ The pathways that connect cachexia and heart failure are unclear. In particular, the extent to which impaired nutritional status might contribute to the deterioration of cardiac function needs investigation. The paucity of animal models of heart failure, that consistently develop cachexia, has impeded progress in this field.

The unfolded protein response (UPR) has been shown to have both protective and pathological roles in the development of heart failure,^3,4^ and also feeding behaviour.^5,6^ The rapid adaptations to diverse stimuli that induce cytosolic and ER stress, such as protein misfolding, loss of metabolites, the production of reactive oxygen species, and DNA damage, are in part instigated by the phosphorylation of (eIF2α). This evolutionarily conserved and vital cellular defense system enables tuning of the level of protein translation and transcriptional reprogramming of the cell called the integrated stress response (ISR). Activation of the ISR leads to the induction of PPP1R15A (also known as GADD34), which binds protein phosphatase 1 (PP1) and G-actin to dephosphorylate P-eIF2α.^7,8^ PPP1R15A is positioned at a critical nexus in the ISR pathway where it participates in a negative-feedback loop, controlling levels of phosphorylated eIF2α and the activation of the ISR and protein translation.

Growth Differentiation Factor, GDF15, is an established biomarker of cellular stress and heart failure.^9^ GDF15 acts through a receptor complex that is expressed solely in the hindbrain, through which it activates neuronal pathways that are perceived as aversive and suppresses food intake.^10^ Reduced food intake has been shown to mediate most of the effects of GDF15 on body weight.^11–13^ GDF15 administration was shown to trigger conditioned taste avoidance in mice.^14,15^ GDF15 expression has been shown to be regulated by the ISR in response to nutritional stress,^14,15^ but the contribution of PPP1R15A in this pathway has not been examined.

Although a clear biomarker of heart failure, it is not known whether GDF15 plays a contributory or protective role in this context. Here we examine the relationship of GDF15 with heart failure and its role in cardiac cachexia, utilizing mice that lack catalytically active PPP1R15A.^16^ In our initial studies examining the role of PPP1R15A in different models of heart failure using bone marrow transfer protocols involving whole body irradiation, we serendipitously identified that PPP1R15A is critical in the prevention of irradiation-induced left ventricular heart failure and associated cardiac cachexia. Hearts from mice lacking PPP1R15A show activation of the ISR, fibrosis, inflammation, and high levels of *Gdf15* expression in heart and GDF15 in the circulation. We provide evidence that blockade of GDF15 activity not only prevents cardiac cachexia but remarkably also slows the worsening of cardiac function. Finally, we also provide evidence that suggests GDF15 may contribute to cachexia in patients with heart failure.

## 2. Methods

### 2.1 Animal studies

All experiments were approved locally and by the Home Office, UK (PPL PP9485757). All procedures conform to the guidelines from Directive 2010/63/EU of the European Parliament on the protection of animals used for scientific purposes. Mice were maintained in a 12 h:12 h light:dark cycle (lights on 07:00–19:00), temperature-controlled (22°C) facility, in specific pathogen-free conditions, with ad libitum access to food (RM3(E) Expanded Chow (Special Diets Services)) and water. Sample sizes were determined based on the homogeneity and consistency of characteristics that were sufficient to detect statistically significant differences in body weight, food intake, and serum parameters between groups. Experiments were performed with animals of both genders. The *Ppp1r15a^ΔC/ΔC^* mouse (originally named *Ppp1r15atm1Dron*)^16^ had been previously backcrossed to achieve 97% C57BL/6 J purity as described.^5^ Experimental cohorts of male *Ppp1r15a^ΔC/ΔC^* and wild-type mice were generated by het × het breeding pairs unless otherwise stated. At the end of the study, all mice were euthanized by rising concentrations of CO_2_ inhalation in their cage (CO_2_ flow rate of 2 L/min for 5 min).

#### 2.1.1 Irradiation and bone marrow transfer

Mice were maintained overnight with Baytril before irradiation were subject to whole-body irradiation with two doses of 5.5 Gy (separated by 4 h) followed by reconstitution with 1 × 10^7^ bone marrow (BM) cells. Baytril was administered for 4 weeks after bone marrow transfer. Mice were monitored by body weight and echocardiography over the experimental period. *Ppp1r15a^ΔC/ΔC^* and wildtype mice were euthanized at the desired time point or for a maximum of 12 weeks. Mice were culled earlier if humane endpoints were approached. Blood was obtained from Vena Cava immediately at sacrifice, plasma separated by centrifugation at 6000xg, and stored immediately at −80°C. Isolated organs were fixed in 4% paraformaldehyde for 24 h at room temperature and then stored in 70% ETOH at 4°C prior to being embedded in paraffin. Alternatively, tissues were fresh frozen on dry ice and kept at −80°C until the day of RNA extraction.

For the study involving the use of anti-GDF15 and isotype control antibodies, 13 −14-week-old *Ppp1r15a*^+/+^*or Ppp1r15a^ΔC/ΔC^* male and female littermates were irradiated with 11 Gy and reconstituted with sex-matched *Ppp1r15a*^+/+^ BM. At 4 weeks post irradiation, *Ppp1r15a^ΔC/ΔC^* male and female mice were randomized into the treatment groups with *n* = 5 of each sex in isotype Ab group and *n* = 5 females, *n* = 6 males in the αGDf15 treated group. The mice were assigned by an independent researcher on the basis of body weight such that the mean body weights of each group were not significantly different. Irradiated *Ppp1r15a*^+/+^ mice were not given any treatment and were monitored by echocardiography only at 9 weeks post-irradiation prior to cull and harvesting of tissue. Anti-GDF15 antibodies (mAB2) and IgG isotype control were generated at Pfizer. mAB2 is a monoclonal antibody that neutralizes GDF15 interaction with its receptor GFRAL. mAB2 or IgG isotype control (3 mg/mL in sterile PBS; 10 mg/kg) were administered via subcutaneous injection once every 3 days from 4 weeks post-irradiation, which was repeated every 3 days until the end of the experiment. Researchers who administered the anti-GDF15 and isotype control antibodies were blinded to the identity of the antibody treatment which was prepared by an independent researcher. Mice were monitored by echocardiography prior to irradiation and weekly from 4 weeks post-irradiation. The capture and analysis of echocardiography data were performed by a researcher that was double blinded to both group allocation and identity of the treatments. *Ppp1r15a^ΔC/ΔC^* mice were euthanized at 9.5 weeks post-irradiation or earlier if mice approached humane endpoints, determined by body weight loss and general welfare indicators. These endpoints were assessed by a researcher who was blinded to the assignment of the treatment groups. After euthanasia, blood was immediately obtained via the Vena Cava and tissues harvested. Gonadal fat pads were dissected and weighed, organs were fixed immediately in 4% paraformaldehyde for 24 h at room temperature and then stored in 70% ETOH at 4°C.

#### 2.1.2 Echocardiography

Two-dimensional and M-mode echo were employed to detect the wall motion, the chamber dimensions, and the cardiac function. Cardiac function was assessed on conscious, un-anaesthetized mice with the Vevo 3100 ultrasound system (VisualSonics, 30 MHz MX400 probe). Echocardiographic M-mode images were obtained from a parasternal short axis view and cardiac function was measured on M-mode images. From two-dimensionally targeted M-mode tracings, left ventricular (LV) end-diastolic (LVEDd) and end-systolic diameter (LVESd), and LV anterior wall thickness (LVAWd) and posterior wall (LVPWd) dimensions were measured using Vevolabs software (Visual Sonics). Cardiac contractile function was assessed by LV fractional shortening (LVFS%), which was calculated as follows: LVFS(%) = (LVEDd–LVESd)/LVEDd) × 100. LV mass was calculated LV Mass (mg) = 1.053 × [(LVIDd + LVPWd + IVSd)^3^ − LVIDd^3^].

#### 2.1.3 SM-ISH RNAscope

Detection of mouse *Gdf15* and *Ppp1r15a* transcripts was performed on Formalin-Fixed Paraffin-Embedded (FFPE) sections using Advanced Cell Diagnostics (ACD) RNAscope® 2.5 LS Reagent Kit-RED (Cat No. 322150), RNAscope® LS 2.5 Probe- Mm-Gdf15 (Cat No. 318528) or RNAscope® LS 2.5 Probe-Ppp1r15a (Cat No. 556038) (ACD, Hayward, CA, USA). Details in Supplementary methods.

##### 2.1.3.1 Spatial plots

*Ppp1r15a;* Cells with an expression score of 1 or more cells were selected and used to generate spatial plots in HALO, in which individual spots represent single cells coloured by expression level from blue (low expression) to red (high expression). *Gdf15*; Cells expression scores were separated into 5 bins of 0, 1–2, 3–5, 6–10 and greater than 10 spots per cell. These data were displayed in HALO using a colour key for the different bin sizes.

#### 2.1.4 Tissue processing and RNA analysis

The fresh heart, liver, and kidney were harvested and snap-frozen in liquid nitrogen. Tissue samples frozen at −80°C were handled on dry ice and 20 mg of the tissue was placed in a pre-cooled 1.5 mL flat-bottom tube containing four Yttria-stabilized (Y57) Grinding Media 5 mm Balls (Inframat Advanced Materials) and 600 uL of Buffer RLT from Qiagen RNeasy Mini Kit. Tissue was subsequently homogenized in a Qiagen® Tissuelyser LT for 4 min at 50 Hz and lysate transferred to 1.5 mL rounded-bottom Eppendorf tube. RNA was extracted with Qiagen RNeasy Mini Kit (no. 74106) and quantified using a Nano drop device.

500 ng of RNA was reverse-transcribed using QuantiTect Rev. Transcription Kit (Qiagen) according to manufacturer’s instruction. Real-time PCR was performed by using SYBR Green qPCR mix (Eurogentec) on a Roche Lightcycler. Alternatively, 500 ng of RNA was used to generate cDNA using Invitrogen SuperScript VILO cDNA Synthesis Kit (Catalog number:11754050) according to manufacturer’s instructions. Gene expression was analysed via TaqMan™ RNA-to-CT™ 1-Step Kit (catalog number: 392653) 2× universal PCR Master mix (Applied Biosystems Thermo Fisher, 4318157) and analysed using 7500 Real time Fast, PCR machine (Applied Biosystems, Thermo Fisher). All reactions were carried out in either duplicate or triplicate and *C*_t_ values were obtained. Relative differences in gene expression were normalized to the expression levels of the housekeeping genes *Hprt or 36B4*. All qPCR primers are shown in Supplementary material online, *Table S1*.

##### 2.1.4.1 Immunofluorescence

Paraffin (PFA) sections were processed as described in supplementary methods.

#### 2.1.5 Measurement of parameters in plasma

In heart failure studies, GDF15 levels were measured using Mouse/Rat GDF15 Quantikine ELISA Kit (no. MGD-150, R&D Systems). Plasma Troponin-I level was measured using the Muscle Injury Panel 3 Mouse Kit (cat# K15186C, Meso Scale Diagnostics). Plasma non-esterified fatty acid concentration was measured using the Roche Free Fatty Acid Kit (half-micro test) (cat#11383175001, Sigma Aldrich). Plasma levels of glucose and triglycerides were measured on a Dimension EXL Analyser (Siemens Healthcare, Erlangen, Germany) using the DF30 and DF69A cartridges (Siemens Healthcare), respectively. Plasma concentration of insulin was measured using the Mouse/Rat Insulin Kit (cat# K152BZC-3; Meso Scale Diagnostics). Plasma corticosterone level was measured by the IDS Corticosterone EIA kit (Immunodiagnostic Systems).

### 2.2 Human studies

#### 2.2.1 Patient population

The BIOlogy Study to TAilored Treatment in Chronic Heart Failure (BIOSTAT-CHF) was an international, multinational, observational study, of which design and primary results have been published previously.^17^ The study protocol conformed to the principles outlined in the declaration of Helsinki and was approved by local and national medical ethics committees. All participants provided written informed consent before study inclusion.

The investigation conformed to the principles outlined in the Declaration of Helsinki and the UK Human Tissue Act 2004. Briefly, patients were included in the in- or outpatient setting, received ≤50% of target dosages of ACEi/ARB and/or beta-blockers at time of inclusion and were anticipated to be uptitrated by the treating physicians. Patients were required to have a left ventricular ejection fraction (LVEF) of ≤40% at inclusion or have plasma concentrations of BNP and/or NT-proBNP >400 pg/mL or >2000 pg/mL, respectively. Out of 2516 patients from the original BIOSTAT-CHF study, GDF-15 levels were available from 2300 patients.

#### 2.2.2 Measurements

Plasma concentrations of GDF-15 were measured using electrochemiluminescence on a cobas e411 analyzer, using standard methods (Roche Diagnostics GmbH, Mannheim, Germany)’. Data on demographics including age, sex, medical history, weight, and height were captured for all patients. Renal function was assessed using the estimated glomerular filtration rate (eGFR) based on serum creatinine. Urine samples were available in 2282 patients from the index cohort. Spot sample urine was stored at −80°C. Protein intake in 24-h urine was calculated by the Maroni method, which was adjusted for the use of spot samples as previously published.^18,19^ The formula used for protein intake in gram/day was as follows: 13.9 + 0.907 * Body mass index (BMI) (kg/m^2^) + 0.0305 * urinary urea nitrogen level (mg/dL). Urinary creatinine was determined in spot-urine samples of 2144 patients. Patients fit the criteria for cachexia if BMI was less than 20 kg/m^2^ and exhibited at least one of the following, CRP > 5 mg/mL, Hb < 12 g/dL or Albumin < 3.2 g/dL.^20^

#### 2.2.3 Statistical analyses

To investigate the association between plasma GDF-15 levels, urinary creatinine, and protein intake as a measure of cachexia, we performed univariable continuous regression with log-transformed GDF15 as the independent variable, and urinary creatinine and estimated protein intake as the independent variables. In subsequent analyses, these were mutually adjusted for possible confounders including age, sex, BMI, log-transformed eGFR, and a medical history of diabetes. All analysis was performed using R (version 3.5).

All numeric data in mice, unless otherwise stated, were analysed using Graphpad Prism version10 (Graph Pad Software, USA). H-score was calculated = Σ (bin number × percentage of cells per bin). Data are represented as arithmetic mean ± SEM or show individual data points with arithmetic mean ±SD. Differences between values were examined using the parametric two-tailed unpaired Student’s *t* test. Means of multiple groups were compared by one way or two-way ANOVA and corrections made for multiple comparison using either Tukey’s, Sidak’s, or Dunnet’s post-tests which was dependent on the comparisons being made and were assigned by Graphpad Prism. All tests were two-tailed. *P* values <0.05 were deemed significant.

## 3. Results

### 3.1 Mice lacking functional PPP1R15A exhibit left ventricular dilated cardiomyopathy and severe weight loss following irradiation

**Figure 1:**
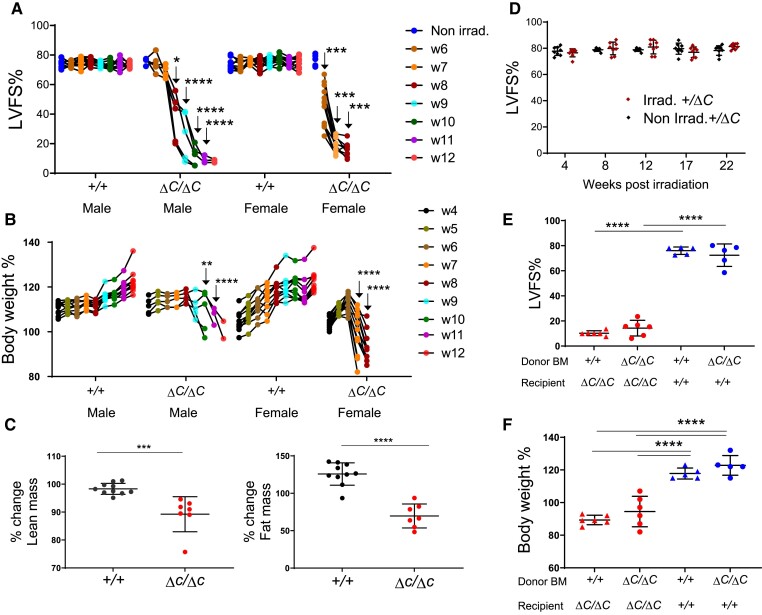
Mice lacking functional PPP1R15A exhibit severe heart failure and weight loss following whole-body irradiation. Mice lacking functional PPP1R15A (^ΔC/ΔC^), male n = 5, or female n = 10, or wild type female (^+/+^) n = 10 and male (^+/+^) n = 10 controls were subjected to whole body irradiation (11 Gy) then bone marrow (BM) transfer and monitored over a time course (weeks 1–12) by (A) echocardiography for left ventricular fractional shortening (LVFS%) and (B) body weight (% of body weight compared to day 0 of irradiation). The number of mice decreased over time due to euthanasia due to reaching humane endpoints. Male (^ΔC/ΔC^) and (^+/+^) mice are littermates. Female mice (^ΔC/ΔC^) n =10, shown from combined experiments, are not littermates. P values of data show comparison between genotypes at identical time points calculated by two-way ANOVA with Sidaks correction for multiple comparisons. Male and female mice were calculated independently (C) TD-NMR analysis of body composition of lean and fat mass using an independent cohort of mice using two-tailed unpaired t test (D) Non-irradiated and irradiated Ppp1r15a^+/ΔC^ littermates heart function (LVFS%) up to 22 weeks. Irradiated Ppp1r15a^+/+^ and Ppp1r15a^ΔC/ΔC^ male mice reconstituted with either Ppp1r15a^+/+^ or Ppp1r15a^ΔC/ΔC^ BM, and show (E) LVFS% and (F) body weight at 9 weeks post-irradiation. e,f using two-way ANOVA with Tukey’s correction. **P < 0.01, ***P < 0.001, ****P < 0.0001.

Following standard protocols of whole-body irradiation at 11 Gy in which mice were reconstituted with bone marrow (BM) immediately after irradiation to replenish the radiation-sensitive immune cells, male and female mice lacking functional PPP1R15A (*Ppp1r15a*^ΔC/ΔC^) and receiving a wild type (WT) BM exhibited an unusual response in that they exhibited a reduction in heart function at 6 weeks post irradiation. Echocardiographic assessment revealed a reduction in left ventricular fractional shortening (LVFS%), culminating in severe heart failure by 7–12 weeks after irradiation (*Figure 1A*). Analysis of heart geometry of the *Ppp1r15a*^ΔC/ΔC^ mice revealed the presence of a dilated cardiomyopathy, with a significant increase in LV internal diameters at systole and diastole and no increase in wall thickness (see Supplementary material online, *Figure S1A*–*D*). Heart failure was associated with cachexia, the severity of this model often requiring euthanasia due to loss of body weight or humane endpoints (*Figure 1B*). This response contrasted with irradiated WT littermates, which did not exhibit a significant reduction of heart function or decrease in body weight (*Figure 1A* and *1B*, see Supplementary material online, *Figure S1A*–D).

Comparison of % change in body composition from 4 weeks post irradiation to 9 weeks post irradiation (or earlier if mice reached human endpoints), using an independent cohort of *Ppp1r15a*^ΔC/ΔC^ and *Ppp1r15a*^+/+^ littermates, showed both lean and fat mass were significantly decreased in *Ppp1r15a*^ΔC/ΔC^ mice (*Figure 1C*). Mice heterozygous for *Ppp1r15a* did not develop heart failure over the 22 weeks post-irradiation (*Figure 1D*). We also examined the role of functional PPP1R15A in BM-derived vs. non-BM-derived compartments. These data show that the BM genotype had no impact on the susceptibility of *Ppp1r15a*^ΔC/ΔC^ or the resistance of WT littermates to irradiation-induced heart failure, changes in heart geometry and cachexia (*Figure 1E* and *F* and see Supplementary material online, *Figure S1E* and *F*), suggesting that loss of PPP1R15A activity in immune cells did not contribute to cardiac cachexia.

### 3.2 Heart failure in irradiated *Ppp1r15a*^ΔC/ΔC^ mice is associated with activation of the ISR

**Figure 2:**
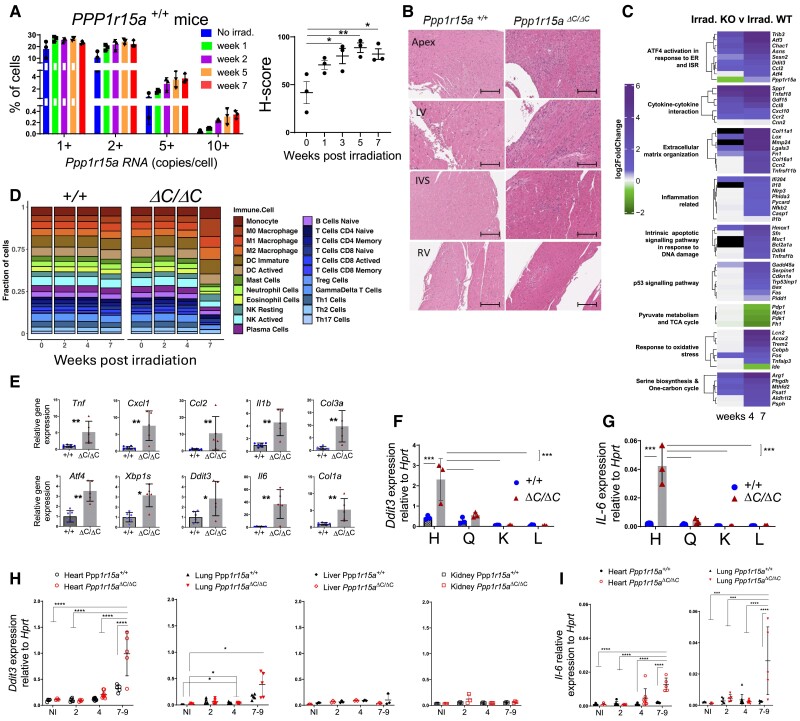
Heart failure is associated with local inflammation and activation of the integrated stress response. (A) Ppp1r15a transcript levels assessed by Single Molecule In Situ Hybridisation (SM-ISH) in heart tissue sections derived from non-irradiated, or irradiated female WT mice, at 13,5 and 7 weeks post-irradiation. H-score of Ppp1r15a expression was analysed by ANOVA and Dunnett’s post-test. (B) Analysis of tissue from Ppp1r15a^+/+^ (^+/+^) or Ppp1r15a^ΔC/ΔC^(^ΔC/ΔC^) male mice (n = 3 mice/group), 7 weeks post-irradiation with H&E staining (scale bar 200 μm). (C) Heatmap of top DEGs related to the selected gene ontology pathways. Comparison between week 4,7 post-irradiation Ppp1r15a^ΔC/ΔC^ (KO) and Ppp1r15a^+/+^ (WT) log2FoldChanges. The Black cell colour represents genes not identified in the corresponding DEGs analysis. (D) Breakdown of immune system cell types found in none and post-irradiation of Ppp1r15a^+/+^ (^+/+^) or Ppp1r15a^ΔC/ΔC^(^ΔC/ΔC^) obtained through RNA-seq sample deconvolution. The number following each group name indicates the days post-irradiation (E) RT-qPCR for inflammatory and ISR gene expression in heart (^+/+^n = 6, ^ΔC/ΔC^ n = 5), P values were determined by two-tailed Student’s t-test. Heart (H), quadricep (Q), kidney (K), and liver (L) tissue taken from male mice at 7 weeks from post-irradiation and analysed for expression for (F) Ddit3 and (G) Il6 (n = 3). Tissue taken from female mice at indicated time post-irradiation analysed for (H) Ddit3 (Heart, Lung, Liver. Kidney) and (I) Il6 gene expression (heart and lung). 7–9 week samples taken at the humane endpoint. Data analysed by two-way ANOVA and Tukey’s post-comparison All data expressed as mean +/-SD. *P < 0.05 **P < 0.01, ***P < 0.001, ****P < 0.0001.

Given that the absence of functional PPP1R15A resulted in severe heart failure, we determined whether *Ppp1r15a* expression was induced in the hearts of irradiated WT mice. We analysed *Ppp1r15a* mRNA abundance using single molecule in situ hybridization (SM-ISH), revealing basal expression of *Ppp1r15a* mRNA in non-irradiated hearts that reached a maximal expression 5–7 weeks post-irradiation (*Figure 2A*). *Ppp1r15a* appeared to be uniformly expressed in all regions of the irradiated WT hearts (see Supplementary material online, *Figure S2A*).

In order to understand the reasons for the heart failure, we examined tissue sections taken from *Ppp1r15a*^ΔC/ΔC^ mice and WT littermates (^+/+^). Analysis of H&E stained sections of hearts from female mice at different time points post-irradiation showed a progressive process of inflammation only in *Ppp1r15a*^ΔC/ΔC^ mice, evident from 5 weeks post-irradiation (see Supplementary material online, *Figure S2B*). H&E stained sections of hearts from *Ppp1r15a*^ΔC/ΔC^ male mice, at 7 weeks post-irradiation, showed foci of myocardial inflammation, predominantly in the apex, LV, and interventricular septum (IVS), with relatively few lesions within the right ventricle (*Figure 2B*). Qualitative assessment of the tissues revealed that there were broad swathes of inflammation in which there was no recognizable myocardium, and notably, neutrophils were inconspicuous on histology. These foci of inflammation were associated with fibrosis as confirmed by Sirius red staining and cross polarization (see Supplementary material online, *Figure S2C* and *D*). Immunostaining showed the inflammatory cells included CD3^+^T cells and LAMP2^+^ macrophages (see Supplementary material online, *Figure S2E* and *F*). These inflammatory changes were not detectable in WT littermates.

To gain a greater understanding of the transcriptional changes occurring over time we performed a whole transcriptome analysis of left ventricular heart tissue of an independent cohort of *Ppp1r15a*^ΔC/ΔC^ and WT littermates, non-irradiated (NI) and at 0-, 2-, 4- and 7-weeks post irradiation. Despite no gross changes in architecture and obvious inflammation, analysis of irradiated WT tissue revealed enrichment of DNA damage, integrated stress response (ISR), inflammation, oxidative stress, and metabolic pathways with irradiation (see Supplementary material online, *Figure S2G* and *Tables S7–S9, S13–S15*). There were no significant differences in gene ontology pathways of *Ppp1r15a*^ΔC/ΔC^ and WT littermates in non-irradiated or 2 weeks post irradiation. However, at 4 and 7-weeks post irradiation there was a significant enrichment of inflammatory pathways in heart tissue from *Ppp1r15a*^ΔC/ΔC^ mice (*Figure 2C* and Supplementary material online, *Tables S5, S6, S10–S12*). Analysis of transcript levels of the UPR, ISR, inflammatory, and fibrotic markers in hearts of *Ppp1r15a*^ΔC/ΔC^ and WT littermates at 7 weeks post-irradiation revealed significant fold changes. One noticeable metabolic pathway that was significantly enriched in irradiated *Ppp1r15a*^ΔC/ΔC^ mice at 7 weeks was for the serine biosynthesis and 1 carbon pathway. This pathway has been previously shown to be activated by ATF4, and can be activated to mitigate cardiomyocyte dysfunction.^21^ Identification of immune system cell types obtained through RNA-seq sample deconvolution indicated increased proportions of myeloid cell subsets in heart tissue of 7-week irradiated *Ppp1r15a^ΔC/ΔC^* mice (*Figure 2D* and see Supplementary material online, *Table S4*).

The increased inflammatory and ISR gene expression in the heart of *Ppp1r15a*^ΔC/ΔC^ mice was corroborated by using an independent cohort of *Ppp1r15a*^ΔC/ΔC^ and WT littermates and performing RT-qPCR from RNA isolated from the heart (*Figure 2E*). Activation of the ISR was indicated by upregulation of *Ddit3* and *Atf4*. Although *Atf4* gene expression can be mediated independently of eIF2α phosphorylation, *Ddit3* gene expression is dependent on eIF2αP and occurs as a result of activation of the ISR.^7^ The increase of XBP-1 splicing (*XBP1*s) indicated activation of the ER stress sentinel, IRE1, and the possible contribution of ER stress-mediated induction of UPR and ISR. In agreement with RNAseq data, inflammatory genes *Tnf*, *Il1b*, *Ccl2*, *Cxcl1*, *Col1a,* and *Col3a* were increased significantly, with the greatest fold change in *Il6* (*Figure 2E*), confirming the histological findings of inflammation and fibrosis in irradiated *Ppp1r15a*^ΔC/ΔC^ mice (see Supplementary material online, *Figure S2B*–*F*).

Using *Il6* and *Ddit3* as indicators of inflammation and ISR activation, respectively, we analysed their expression in a panel of tissues, heart, quadriceps, kidney, and liver taken from *Ppp1r15a*^ΔC/ΔC^ and WT littermates at 7 weeks post irradiation (*Figure 2F* and *G*). ISR activation and *Il6* gene expression were upregulated in the hearts of mice lacking PPP1R15A activity and was not evident in skeletal muscle kidney or liver. In a separate cohort of female mice, heart, lung, kidney, and liver tissue was harvested over the irradiation time course (*Figure 2H*). Activation of the ISR was evident at the late stage of the disease in the heart with a smaller induction in lung but not kidney and liver. *Il6* upregulation was also evident in heart and lung, but not other organs tested (*Figure 2I*).

### 3.3 *Ppp1r15a* ^ΔC/ΔC^ cardiomyocytes express high levels of GDF15 after irradiation

**Figure 3:**
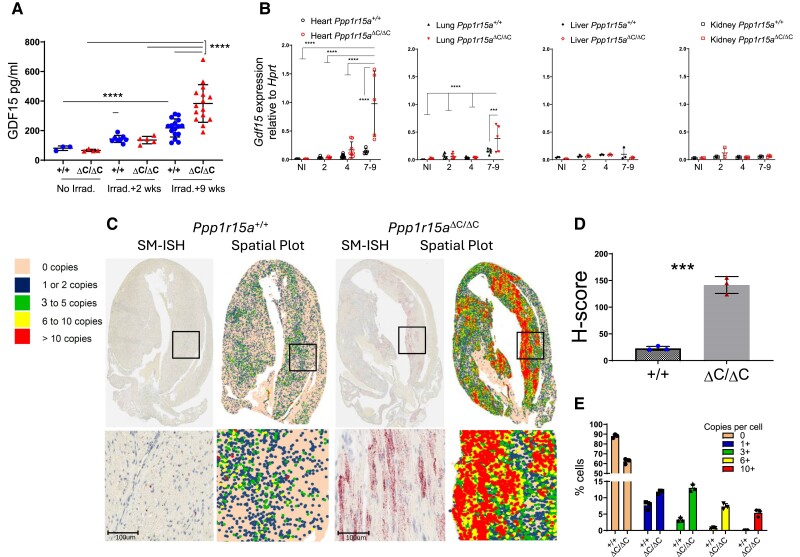
Absence of functional PPP1R15A results in increased expression of *Gdf15* following whole-body irradiation. *Ppp1r15a^ΔC/ΔC^(^ΔC/ΔC^)* and *Ppp1r15a*^+/+^ (*^+/+^*) mice were irradiated (11 Gy) and reconstituted with *Ppp1r15a*^+/+^ bone marrow. (*A*) GDF15 in plasma from non-irradiated mice, and at 2 and 9 weeks post-irradiation. (*B*) *Gdf15* expression, by RT-qPCR, in heart, lung, liver and kidney taken at 7 weeks post-irradiation. *P* values by two-way ANOVA with Tukey’s correction. *Gdf15* expression in hearts, assessed by SM-ISH. (*C*) Representative spatial plots, (*D*) H-score (*E*) and the distribution of cells expressing *Gdf15* transcripts, *n* = 3 hearts/group. % cells and H-score are mean ± SD (*P* values by students *t* test). ***P < 0.001, ****P < 0.0001.

One candidate that may drive the cachexia in this model is a recently described peptide hormone, GDF15, whose induction by cellular stress and the ISR has been well described and acts centrally in suppressing food intake.^22,23^ GDF15 can only mediate its effects centrally through increased levels in the circulation.^24^ Therefore, we examined plasma GDF15 from *Ppp1r15a*^ΔC/ΔC^ and *Ppp1r15a*^+/+^ mice, non-irradiated, and at different time points post-irradiation. Plasma GDF15 was elevated following irradiation at 9 weeks post-irradiation in mice of both genotypes, but *Ppp1r15a*^ΔC/ΔC^ showed significantly higher levels (*Figure 3A*).

In order to identify the source of GDF15 in the irradiated mice, we analysed *Gdf15* mRNA expression in heart, kidney, liver, and lung, which identified the heart as potentially the major contributor to the increased GDF15 in plasma of *Ppp1r15a^ΔC/ΔC^* mice (*Figure 3B*). To identify the cells expressing *Gdf15*, we utilized SM-ISH by RNAscope on tissue sections prepared from male *Ppp1r15a^ΔC/ΔC^* and WT hearts at 7 weeks post-irradiation (*Figure 3C*). G*df15* mRNA was highly expressed in cardiomyocytes, identified by their morphology and presence of striations, in *Ppp1r15a^ΔC/ΔC^* hearts, and significantly greater than irradiated WT mice when quantified by copies per cell and H-score (*Figure 3D* and *E*). *Gdf15*-expressing cardiomyocytes were abundant in apex, LV, and IVS, while relatively sparse in the right ventricles of *Ppp1r15a^ΔC/ΔC^* mice. Typically, *Gdf15* expression was highest in cardiomyocytes that were proximal to the inflammatory lesions. In contrast, inflammatory cells did not express conspicuous *Gdf15*. Analysis of hearts taken at different time points post-irradiation revealed a gradual increase in *Gdf15* expression over time, single cardiomyocytes expressing high quantities of *Gdf15* in the absence of inflammatory foci were detectable as early as 3 weeks post-irradiation (see Supplementary material online, *Figure S3*). This suggests that cardiomyocyte GDF15 expression, an indicator of cellular stress, may precede the infiltration of the inflammatory cells in *Ppp1r15a^ΔC/ΔC^* mice.

### 3.4 The absence of CHOP does not prevent irradiation-induced cardiac cachexia nor GDF15 expression

**Figure 4:**
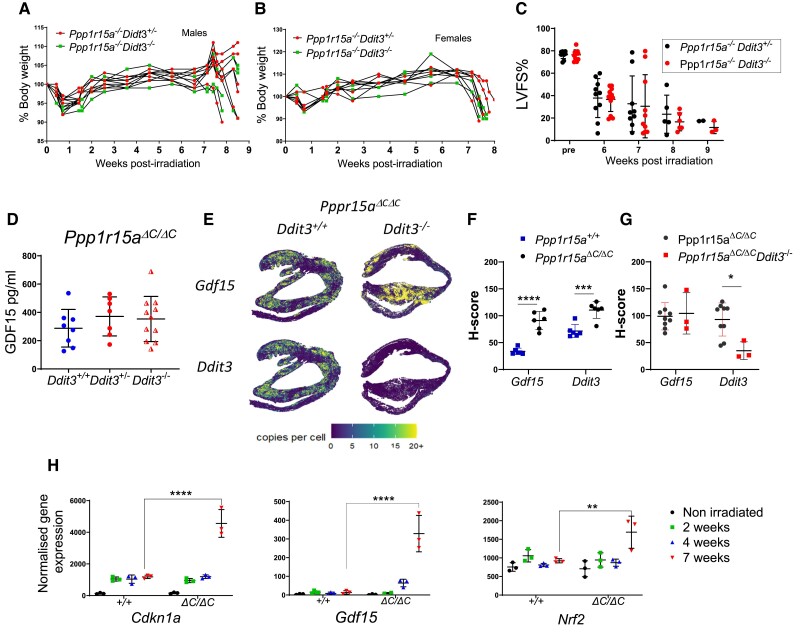
The absence of *Ddit3* does not prevent irradiation-induced heart failure or GDF15 expression. *Ppp1r15a^ΔC/ΔC^(^ΔC/ΔC^) Ddit3^+/−^, or Ppp1r15a^ΔC/ΔC^(^ΔC/ΔC^) Ddit3^−/−^ mice, n = 10 per group* were irradiated (11 Gy) and reconstituted with *Ppp1r15a^+/+^* BM. Mice were culled at 8.5 weeks post-irradiation or earlier due to humane endpoints. % of body weight of (*A*) male and (*B*) female mice. (*C*) LV function (LVFS %) assessed by echocardiography. (*D*) GDF15 analysis of the plasma and comparison with irradiated *Ppp1r15a^ΔC/ΔC^(^ΔC/ΔC^) Ddit3^+/+^ mice. Gdf15* and *Ddit3* expression in heart tissue assessed by SM-ISH and distribution was shown in (*E*) spatial plots and (*F*, *G*) H-score of cells expressing *GDF15 and Ddit3* transcripts. H-score was analysed by ANOVA and Dunnett’s post-test. (*H*) Normalized gene expression from RNAseq analysis for *P* values determined by two-way ANOVA. Data show mean ± SD. **P < 0.01, ***P < 0.001, ****P < 0.0001.

CHOP (encoded by *Ddit3*) is a transcription factor highly induced during the UPR and deletion of the *Ddit3* gene has provided protection against tissue damage and disease, including atherosclerosis^25^ and renal damage caused by the UPR-inducing toxin tunicamycin.^26^ We therefore examined whether absence of *Ddit3* impacted on development of irradiation-induced heart failure and weight loss. *Ppp1r15a^ΔC/ΔC^* mice lacking *Ddit3* showed no protection from irradiation-induced weight loss or heart failure (*Figure 4A–C*). CHOP has also been described as an important factor in inducing GDF15 expression following induction of the ISR^27^ however, the absence of CHOP (*Ddit3)* had no significant impact in the levels of GDF15 detected in plasma nor expression in heart tissue (*Figure 4D–G*). NRF2 and p53 have been demonstrated as transcription factors in the induction of GDF15.^28,29^ We show enrichment of p53 pathways (*Figure 1C*), including activation of *Cdnkn1a*, and significant induction of *Nrf2* gene expression in *Ppp1r15a^ΔC/ΔC^* hearts at 7 weeks post irradiation (*Figure 4H*). This raises the possibility that these factors may be involved in regulation of GDF15 in this model.

### 3.5 Blockade of GDF15 activity prevents weight loss and cachexia and slows the worsening of cardiac function

**Figure 5:**
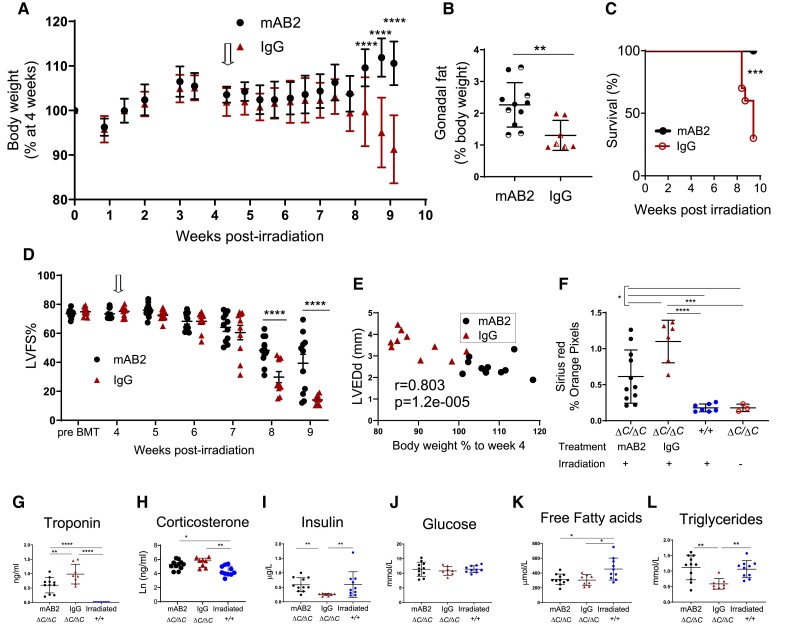
GDF15 is required for weight-loss and severe heart failure in irradiated mice lacking functional PPP1R15A. *Ppp1r15a^ΔC/ΔC^* or *Ppp1r15a*^+/+^male and female mice were irradiated (11 Gy) and reconstituted with *Ppp1r15a^+/+^* BM. At 4 weeks (open arrow), *Ppp1r15a^ΔC/ΔC^* mice were randomly assigned and given an antibody that blocks GDF15 activity (αGDF15), *n* = 11 or an isotype control antibody (Isotype Ab), *n* = 10, at 10 mg/kg, at 3-day intervals. Mice were culled at 9.5 weeks post-irradiation or earlier due to humane end points. (*A*) Body weights, % of body weight at 4 weeks post-irradiation. *P* values were determined by two-way ANOVA with Sidaks correction for multiple comparisons, *P* values indicated by asterisks, show comparison between treatment groups at identical time points. (*B*) Relative weight of fat pads, female mice indicated by half-filled symbols. *P* values were calculated by two-tailed Student’s *t*-test. (*C*) Survival plot, no mice were found dead but mice that approached humane endpoints were euthanized. Data analysed using Gehan-Breslow-Wilcoxon Test. (*D*) LV function (LVFS %) was assessed by echocardiography at 9 weeks post-irradiation. Data were analysed using ANOVA and Tukey’s post comparison. (*E*) Correlation of LV end diameter at diastole (LVEDd) with % body weight at 9 weeks post irradiation, *r* calculated by Pearson’s correlation coefficient. (*F*) Analysis of fibrosis in heart tissue sections. Blood taken at 9.5 weeks post-irradiation (or earlier due to humane endpoints) and plasma analysed for (*G*)) troponin and (*H–L*) metabolic parameters as indicated. Data (*F–H*, *I*, *K*, *L*) analysed using one way ANOVA and Tukey test post comparison. Data in (*J*) analysed using Kruskal-Wallis test with Dunn’s post test. All data show mean ± SD. *P < 0.05 **P < 0.01, ***P < 0.001, ****P < 0.0001.

Given the increased GDF15 in irradiated *Ppp1r15a^ΔC/ΔC^* mice, we hypothesized that the cachexia was mediated by reduced food intake via GDF15 signalling. In order to test this hypothesis, we used a monoclonal antibody mAB2, against mouse GDF15. This monoclonal antibody has been validated to block the functional activity of GDF15, whereby mAB2 rapidly reversed the weight loss induced by recombinant GDF15 as opposed to mice receiving an isotype control.^30^ To investigate whether GDF15 was driving cachexia we analysed whether mAB2 could inhibit loss of body weight in irradiated *Ppp1r15a***^ΔC/ΔC^** male and female mice. mAB2 or IgG were administered at the beginning of week 4 post-irradiation and repeated at 3-day intervals until the end of week 9. Mice treated with control IgG exhibited severe weight loss from 8 weeks onward, which was in striking contrast to mice receiving mAB2 (*Figure 5A*), suggesting that the weight loss in this model was GDF15-mediated. The loss in body weight in the control group was, in part, attributed to loss of fat mass, indicated by the lower gonadal fat pad weight in the IgG-treated group (*Figure 5B*). The difference in severity of the treatment groups was highlighted by the finding that only mice in the IgG-treated group required euthanasia, due to a combination of body weight loss and other welfare indicators (*Figure 5C*).

Unexpectedly, blocking GDF15 activity also attenuated the development of severe heart failure. GDF15 blockade significantly prevented the decrease in LV function compared to the IgG-treated group (*Figure 5D* and *E*). This was also shown by differences in other parameters of heart function (see Supplementary material online, *Figure S4A*–*G*). Heart fibrosis and plasma troponin a measure of cardiac damage, were also reduced by mAB2 (*Figure 5F* and *G*). The strong correlation of body weight with heart function suggests that these parameters are interconnected in this model (*Figure 5E*). Furthermore, we show a significant correlation of GDF15 plasma concentration and body weight (see Supplementary material online, *Figure S4H*), supporting the idea that the increased levels of GDF15 in *Ppp1r15a***^ΔC/ΔC^** mice are biologically relevant. Activation of the hypothalamic-pituitary-adrenal stress axis was evident by the increase in corticosterone in *Ppp1r15a*^ΔC/ΔC^ mice compared to irradiated WT mice (see Supplementary material online, *Figure S5H*), which ruled out the possibility of adrenal gland dysfunction as a cause of weight loss. GDF15 can also the stimulate the hypothalamic-pituitary-adrenal axis in response to a range of stressful stimul.^31^ Given that we show no significant difference in corticosterone levels in the presence of GDF15 blocking antibody in this mode, this would suggest that this property of GDF15 does not contribute to this heart failure model. Analysis of other metabolic parameters (*Figure 5I–L*) showed that glucose levels in the blood were sustained, with insulin levels significantly reduced in IgG-treated mice. Free fatty acids (FFAs) were significantly lower in *Ppp1r15a*^ΔC/ΔC^ mice compared to irradiated WT mice, with a reduction in plasma triglycerides only in IgG treated *Ppp1r15a*^ΔC/ΔC^ mice. These data suggest that GDF15 signalling pathways contribute towards cardiac cachexia in this model and impact significantly on the development of the dilated cardiomyopathy. Protective effects on body weight and heart function were also observed in irradiated mice that lacked *Ppp1r15a***^ΔC/ΔC^** and GDF15 compared to *Ppp1r15a***^ΔC/ΔC^** GDF15^+/+^ mice (see Supplementary material online, *Figure S4I* and *J*).

### 3.6 GDF15 expression correlates with parameters of heart function in another murine model of cardiac cachexia

We also examined GDF15 expression in a murine model of dilated cardiomyopathy where cardiac-specific ablation of Yme1l (cYKO) in mice induces mitochondrial fragmentation and altered cardiac metabolism. These mice have been previously shown to exhibit dramatic weight loss at approximately 42 weeks of age.^32^ We measured plasma GDF15 in a cohort of these mice that exhibited a reduction in heart function at 34–35 weeks of age. At this stage, it was not expected that all mice would be at the final stage of the disease, although a proportion of the mice exhibited rapid weight loss (see Supplementary material online, *Figure S5A* and *B*). Plasma GDF15 correlated strongly with changes in parameters of heart geometry and heart function (see Supplementary material online, *Figure S5C*–*E*).

### 3.7 GDF15 expression correlates with cachexia in a cohort of patients with heart failure

**Figure 6:**
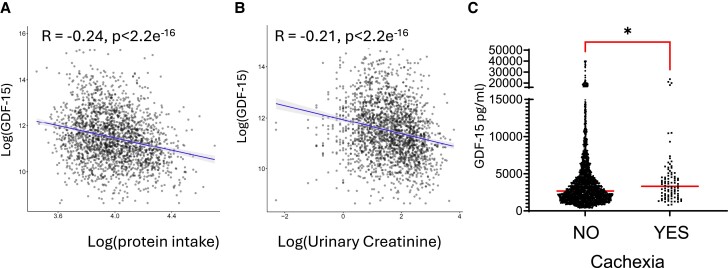
GDF15 correlates with parameters of cachexia in patients with heart failure. Analysis of the BIOSTAT-CHF cohort of patients with heart failure, the correlation between Log-transformed plasma GDF-15 and (*A*) log-transformed estimated protein intake *n* = 2112 and (*B*), log-transformed urinary creatinine. *n* = 2143. (*C*) Dot plots of plasma GDF15 were compared in patient groups according to whether they fit the criteria for cachexia. No *n* = 2153, or Yes *n* = 90. Data analysed using Mann–Whitney *U* test *P < 0.05. Medians are shown.

Weight loss is strongly associated with adverse outcomes in patients with chronic heart failure.^33^ In addition, GDF15 has been identified as an important predictor of mortality in patients with heart failure.^9^ To investigate the association between GDF15 and cardiac cachexia we analysed the BIOSTAT-CHF cohort, which included 2516 patients with worsening signs and/or symptoms of heart failure.^17^ Protein intake in 24-h urine was calculated by the Maroni formula. Since spot samples were used, the adjusted Maroni formula was utilized. Plasma GDF-15 levels were inversely correlated with protein intake (*Figure 6A*). The concentration of serum creatinine, produced after stable conversion of creatine largely found in skeletal muscles, can also be used as a marker to reflect (peripheral) muscle catabolism and is an established marker of muscle mass^34^ and can be used as a measure of cachexia.^35,36^ The adjusted formula and use of spot urine samples was validated in a heart failure population using data from the Additive renin Inhibition with Aliskiren on renal blood flow and Neurohormonal Activation in patients with Chronic Heart Failure and Renal Dysfunction cohort (ARIANA-CHF-RD).^37^ GDF15 inversely correlated with urinary creatinine (*Figure 6B*). These associations remained statistically significant for both protein intake (standardized β: −0.20, *P* < 0.001) and urinary creatinine (β: −0.18, *P* < 0.001) in multivariable regression analyses mutually adjusted for age, sex, BMI, log-transformed eGFR and medical history of diabetes. Furthermore, patients that fitted the criteria for cachexia, exhibited greater plasma GDF15 than those that did not fit the criteria (*Figure 6C*).

## 4. Discussion

We identified PPP1R15A as an essential factor in protection from radiation-induced heart failure and associated cardiac cachexia. The mice lacking functional PPP1R15A exhibited a dilated cardiomyopathy histologically characterized by loss of cardiomyocytes, a prominent immune cell infiltration, fibrosis and pro-inflammatory cytokine expression.

The presence of inflammatory infiltrates and the enrichment of inflammatory gene ontology pathways at 4 weeks post irradiation in mice lacking PPP1R15A activity, suggests this is a precursor to heart dysfunction. Cardiomyopathy developed irrespective of the PPP1R15A status of BM-derived cells, suggesting that the loss of PPP1R15A in bone marrow-derived immune cells were not critical in this heart failure model. The finding that the inflammation, *Gdf15* and ISR activation predominated in heart and not skeletal muscle, nor kidney and liver suggests that heart tissue is intrinsically vulnerable to irradiation in the absence of PPP1R15A. GDF15 expression is associated with mitochondrial dysfunction and cellular stress and is clearly evident in the cardiomyocytes of *Ppp1r15a^ΔC/ΔC^* mice at 7–9 weeks post irradiation. The cardiomyocyte cellular stress may be the primary dysfunction or secondary to perturbation of other cell types, such as capillary endothelial cells which are radiation sensitive.^38^ The predominance of myeloid cells and fibrosis is similar to human myocarditis, where monocytes and macrophages predominate in the myocardium infiltrate.^39^

PPP1R15A functions in its canonical role to limit the extent of ISR activation and reversal of stress-induced translational attenuation.^7,40,41^ In support of this, we show evidence of enhanced ISR activation in *Ppp1r15a^ΔC/ΔC^* mice in heart tissues but this only occurred at 7–9 weeks post irradiation when weight loss and heart dysfunction was evident. This suggests that a pivot point may exist where the loss of PPP1R15A dysregulated ISR drives cell injury and most likely death, and an inflammatory cascade. In tissue culture PPP1R15A (GADD34) mutant cells are more prone to cell death in response to ER stress induced by thapsigargin.^7^ This agent causes ER stress and induction of the UPR and ISR through depletion of ER calcium stores and is particularly effective at shutting down protein synthesis, indicating that recovery of protein translation is necessary for the adaptive pro-survival effects.

This protective role of PPP1R15A shown here is counter to studies where mice lacking PPP1R15A activity are protected from UPR-induced nephrotoxicity^16^ suggesting that specific organs may have different requirements for sustaining protein synthesis in the face of ISR activation. The use of small molecules that mimic the effect of PPP1R15A activity by preventing translational shut down,^42^ are attractive candidates to utilize *in vivo* to test the hypothesis that PPP1R15A activity protects WT mice from heart failure by preventing over-exuberant translation attenuation and ISR activation.

One consistent feature in the irradiated *Ppp1r15a^ΔC/ΔC^* mice was the prominent fibrosis of the left ventricle at the later stages of the disease. A previous study examining wound healing, showed that mice lacking PPP1R15A exhibited accelerated wound closure, increased number of myofibroblasts, and elevated collagen production.^43^ A recent report has also shown that mice lacking *Ppp1r15a* show increased pulmonary fibrosis in response to the DNA damage-inducing chemical bleomycin.^44^ Taken together, these data suggest that the PPP1R15A may play an important role in the negative regulation of fibrosis pathways.

The protective effects of anti-GDF15 treatment in heart failure are in contrast to the findings that GDF15-deficient mice were more susceptible to cardiac rupture in a murine model of acute myocardial infarction.^45,46^ In a report by Xu *et al*.,^47^ genetic deletion of Gdf15 promoted LV hypertrophy and systolic dysfunction after transverse aortic constriction. These discrepancies may result from the fact that heart failure is induced by different mechanisms from the studies reported here. These reports suggested that GDF15 acts directly on neutrophils and cardiomyocytes but it remains unknown whether those cells express the GDF15 receptor, GFRAL/Ret. The expression of the latter has been shown to be restricted to the postrema area of the brainstem. It remains possible, however, that GDF15 signals through other receptor(s)/mechanism(s) outside the brainstem. Most importantly cachexia was not apparent in these models, so the role of GDF15 was not examined in this context.

More recently, Luan *et al*. found that anti-GDF15 treatment resulted in increased cardiac damage following injection of LPS over 48 h.^48^ A caveat of that study was that an anti-human GDF15 was used to block endogenous murine GDF15, while some evidence was presented on tissue sections showing its ability to attenuate murine GDF15 signalling, no data was provided to show the efficacy of this antibody in the prevention of weight loss induced by the recombinant GDF15. These findings are contradicted by a study using a validated blocking antibody raised against murine GDF15, where no effect was shown in lethality or weight loss following high or low dose LPS treatment.^30^

The hindbrain receptor for GDF15 has recently been identified and the action by its ligand, powerfully suppresses food intake.^11–13^ The neutralization of GDF15 in our model substantially slowed the development of weight loss. We assume this occurred by a mechanism that at least in part involved an improvement in food intake, a limitation in the study was that we were unable to undertake single housing experiments necessary to measure this directly. Our analysis of metabolites in the circulation suggests that the most cachectic mice had significantly lower nutrient stores and that blocking GDF15 maintains food intake and triglyceride levels. The dynamic changes in cardiomyocyte-expressed GDF15 in *Ppp1r15a^ΔC/ΔC^* match other markers of ISR activation and cardiomyocyte dysfunction. Although the GDF15 in serum increased by only 2-fold compared to irradiated WT mice, the levels of GDF15 are biologically relevant. This relevance was strikingly shown, where inhibition of GDF15 activity had a substantial impact on the development of severe heart failure and the necessity for euthanasia. The context of GDF15 expression may be a critical factor in this model. Analysis of the effect of recombinant GDF15 on irradiated WT mice would reveal whether raised GDF15 levels was a dominant factor. We show that blocking GDF15 reduced damage to the heart but also reduced heart fibrosis, and we presume this protection occurs downstream of GDF15 signalling in the hind brain although the pathway for this protection needs further investigation. It is not known whether the loss of GDF15 signalling mediates its cardio-protection via its effects on body weight, intake of food that provides vital fuel substrates for the heart, or additional signalling circuits emanating from the hindbrain that mediate cardio-protective effects. Studies in mice using murine models of cancer cachexia, show that GDF15 neutralization inhibits cachexia^49^ and improves muscle function and physical performance^50^ and suggested that the improvements were due to increased calorie intake and calorie-independent mechanisms.

The strong correlations of GDF15 in plasma and parameters of heart function in the mice lacking *Yme1l* and exhibiting dilated cardiomyopathy, suggest that GDF15 may have broad applicability in driving the severity of heart failure in cardiac cachexia.

In a recent rat model of monocrotaline-induced right-ventricular dysfunction, blocking GDF15 restored the ability of rats to gain weight over the treatment period but had no effect on heart function. It is important to note that this model was not able to test the effect of blocking GDF15 on heart function in the context of cardiac cachexia because the rats did not lose weight over the experimental period.^51^

Using a cohort of patients with heart failure, we showed that GDF15 measured in plasma correlated with surrogate markers in urine for protein intake and muscle mass. Although the study utilized spot urine samples rather than a 24 h urine sample, our recent study using spot urine analysis showed that urinary creatinine concentrations were associated with smaller body dimensions (lower BMI, height, and weight) and an increased risk of (*>*5%) weight loss at 9 months.^37^ The inverse correlation of muscle mass and protein intake with GDF15 and raised GDF15 levels in patients with cachexia, support the concept that GDF15 plays a role in driving cardiac cachexia. Further analyses would be required to determine if GDF15 reduced protein appetite specifically or whether this reduction reflected a reduction in total food intake. It is likely that many factors contribute to cardiac cachexia and may be relevant to particular subgroups of patients. More in-depth analyses may reveal the relationships between changes in GDF15 levels and changes in parameters of cachexia and heart remodelling.

In summary, our work identifies GDF15 as a critical driver of cardiac cachexia and critically impacted on heart function. Although the PPP1R15A deficiency/irradiation model is a novel model of heart failure, it is typified by endoplasmic reticulum stress, mitochondrial dysfunction, and inflammation, which, beyond inflammatory cardiomyopathies, are common features of human heart failure (of both ischemic and non-ischemic origin), as extensively reviewed previously^52,53^ The high GDF15 production and occurrence of cardiac cachexia within a relatively short timeline are most likely due to the severity of our model in terms of ER stress and mitochondrial dysfunction. The other mouse models of heart failure do not appear to be severe enough in terms of cellular stress and mitochondrial dysfunction to induce high GDF15 production and lead to cardiac cachexia. Our findings warrant further studies to investigate the role of GDF15 in human heart failure. Blockade of GDF15 could constitute a novel therapeutic option to limit cardiac cachexia and improve cardiac function and clinical outcomes in patients with severe systolic heart failure. A phase 2 randomized clinical trial is currently assessing the effect of ponsegromab (a monoclonal anti-GDF15 neutralizing antibody) on frequency, severity, and burden of symptoms as well as physical limitations in participants with heart failure and elevated circulating GDF-15 concentrations (NCT05492500).

**Translational perspective**

Cachexia is associated with chronic heart failure and its occurrence independently predicts increased morbidity and mortality independently of other variables. The pathways that connect cachexia and heart failure are unclear. These data using an animal model of cardiac cachexia suggest that blockade of GDF15 would provide a novel therapeutic option to limit cardiac cachexia and improve clinical outcomes in patients with severe systolic heart failure.

## Supplementary Material

cvae214_Supplementary_Data

## Data Availability

All RNASeq analysis scripts, with details of software versions, expression raw count files and results are freely available from https://github.com/CAD-ZM-BFX/Takaoka_Mallat_Goodall (DOI:10.5281/zenodo.13142209).
