# Supplementary material for: GDF15 antagonism limits severe heart failure and prevents cardiac cachexia
Source: Cardiovasc Res. 2024 Sep 23;120(17):2249–60. doi: 10.1093/cvr/cvae214 (PMC11687397; doi:10.1093/cvr/cvae214)
Supplement: cvae214_Supplementary_Data [file cvae214_supplementary_data.zip › Takaoka supplementary methods figures.pdf]

Title: GDF15 antagonism limits severe heart failure and prevents cardiac cachexia

Minoru Takaoka[1†], John A.Tadross[2,3], Ali B.A.K. Al-Hadithi[1], Xiaohui Zhao[1], Rocío Villena-Gutiérrez[4], Jasper Tromp[5,6], Shazia Absar[1], Marcus Au[1], James Harrison[1], Anthony, P. Coll[2]. Stefan J. Marciniak[7], Debra Rimmington[2], Eduardo Oliver[4,8,9], Borja Ibáñez[4,8,10], Adriaan, A. Voors[5], Stephen O'Rahilly[2], Ziad Mallat[1,11,*] and Jane C. Goodall[1,* †]

Short title: GDF15 and heart failure

[1] Victor Phillip Dahdaleh Heart and Lung Research Institute. Department of Medicine, University of Cambridge, Cambridge, UK

[2]Wellcome-MRC Institute of Metabolic Science and Medical Research Council, Metabolic Diseases Unit, University of Cambridge, Cambridge, UK

[3]Department of Histopathology and East Midlands & East of England Genomic Laboratory

[4] Centro Nacional de Investigaciones Cardiovasculares (CNIC), Madrid. Spain.

[5]University of Groningen, University Medical centre Groningen, the Netherlands

[6] Saw Swee Hock School of Public Health, National University of Singapore & the National University Health system, Singapore.

[7]Cambridge Institute for Medical Research, Cambridge Biomedical Campus, University of Cambridge, Cambridge, UK

[8]Centro de Investigaciones Biomédicas en Red de Enfermedades Cardiovasculares (CIBERCV), Madrid. Spain.

[9]Centro de Investigaciones Biologicas Margarita Salas (CIB-CSIC), Madrid, Spain.

[10] IIS-Hospital Fundacion Jimenez Diaz, Madrid, Spain.

[11] Paris Cardiovascular Research Center, Université Paris Cité, INSERM UMRS 970, Paris, France.

[*] Co-senior authors.

[†] Corresponding authors

Jane C. Goodall. jcg23@medschl.cam.ac.uk Minoru Takaoka mt709@cam.ac.uk

Supplementary methods

Study approvals

All studies in U.K. were performed in accordance with UK Home Office Legislation regulated under the Animals (Scientific Procedures) Act 1986 Amendment, Regulations 2012, following ethical review by the University of Cambridge Animal Welfare and Ethical Review Body (AWERB)

Age and sex of mice used in the study

Data in Figure 1a-d are from *Ppp1r15a*$^{ΔC/ΔC}$ male mice and male and female wildtype littermates irradiated at 7-8 weeks old. Data in Figure S1e,f are from *Ppp1r15a*$^{ΔC/ΔC}$ mice irradiated at 11-12 weeks old. Data in Figure S2b are from female *Ppp1r15a*$^{ΔC/ΔC}$ and wildtype littermates irradiated at 10-11 weeks old. Data shown in Figure 2a is from wildtype C57BL/6 mice irradiated at 9 weeks old.

***Ppp1r15a*$^{ΔC/ΔC}$ *Gdf15*$^{-/-}$ mice**

Ppp1r15a$^{ΔC/ΔC}$ *Gdf15*$^{-/-}$ mice were generated by crossing C57BL/6N-Gdf15tm1a(KOMP)Wtsi/H (known as GDF15$^{-/-}$) [1] with *Ppp1r15a*$^{ΔC/ΔC}$ mice to create an F1 *Ppp1r15a*$^{ΔC/ΔC}$ GDF15$^{+/-}$ heterozygote line. These heterozygote mice were used to create the experimental cohorts of *Ppp1r15a*$^{ΔC/ΔC}$ *Gdf15*$^{-/-}$ mice and *Ppp1r15a*$^{ΔC/ΔC}$ *Gdf15*$^{+/+}$ mice.

Dilated cardiomyopathy model using *Yme1l*$^{-/-}$ mice

The cardiac-specific *Yme1l*$^{-/-}$ mice (cYKO) were generated by cross-breeding *Yme1l*$^{LoxP/LoxP}$ flanking exon 3 mice with mice expressing Cre-recombinase under control of the *Myh6* promoter as previously described [25]. Adult mice were maintained under pathogen-free conditions in a temperature-controlled room and a 12-hour light-dark cycle at the CNIC animal facility. Chow diet and water were available ad libitum. Animal experiments conformed to European Union Directive 2010/63EU and Recommendation 2007/526/EC,

enforced in Spanish law under Real Decreto 1386/2018. All experiments were approved for CNIC ethics committee and the Animal Protection Area of the Comunidad de Madrid (PROEX 176.3/20). Echocardiography was performed using the Vevo 2100 and using anaesthesia.

Excluded samples. No sample or animal data were excluded from the data sets unless there was a collection failure. 3 fat pad data points are absent from the isotype Ab data set (Figure 5b) as these mice had deteriorated rapidly and were culled by a technician who did not remove and weigh the fat pads. Data from 2 plasma samples were excluded from isotype Ab treated data set (Figure 5g-i), due to inconsistent sample collection where 2 blood samples were left overnight at room temperature rather than immediate plasma isolation and -80°C freeze.

Immunofluorescence.

Paraffin (PFA) sections (stored at 4°C) were dried for 30 minutes, before being rehydrated in PBS for 10 minutes before the staining. PFA-fixed sections were then permeabilised in 0.1 % Triton X-100, 0.1 % Citrate buffer pH 6.0 (Dako) for 30 minutes. They were then washed in PBS, and incubated with the blocking solution (flow buffer + 5% serum of secondary antibody species, i.e. goat or donkey) for 30 minutes before being incubated with primary antibodies diluted in the blocking solution at indicated concentrations (See extended data table) overnight at 4°C. Samples were extensively washed with PBS and incubated with secondary antibodies diluted in the blocking solution at indicated concentrations (shown in key resources table) for 4 hours. Samples were again washed extensively in PBS, nuclei were counterstained with Hoechst 33342 (Invitrogen) and samples were mounted with CC mountTM (Sigma). Interstitial collagen was detected using picrosirius red staining followed by microscopy under polarized light.

RNAscope

Briefly, sections were cut at 10μM thick, baked for 1 hour at 60ºC before loading onto a Bond RX instrument (Leica Biosystems). Slides were deparaffinized and rehydrated on board before pre-treatments using Epitope Retrieval Solution 2 (Cat No. AR9640, Leica Biosystems) at 95°C for 15 minutes, and ACD Enzyme from the LS Reagent kit at 40ºC for 15 minutes. Probe hybridisation and signal amplification was performed according to the manufacturer's instructions. Fast red detection of mouse *Gdf15* and *Ppp1r15a* was performed on the Bond Rx using the Bond Polymer Refine Red Detection Kit (Leica Biosystems, Cat No. DS9390) according to the ACD protocol. Slides were then removed from the Bond Rx and were heated at 60ºC for 1 hour, dipped in Xylene and mounted using EcoMount Mounting Medium (Biocare Medical, CA, USA. Cat No. EM897L). The slides were imaged on the Aperio AT2 (Leica Biosystems) to create whole slide images. Images were captured at 40x magnification, with a resolution of 0.25 microns per pixel.

RNAseq analysis

RNA-Seq, 50bp paired-end, libraries were generated from defined time points (N=6 for each time point, Supplementary Table 1) with different conditions (WT and KO). Raw fastq data have been deposited in ArrayExpress with accession number E-MTAB-12831(https://www.ebi.ac.uk/biostudies/arrayexpress/studies/E-MTAB-12831).

For each library, original reads files were aligned to GRCm39 mouse genome and quality controlled by using a nextflow (v21.05.0.edge) [2] pipeline nf-core/rnaseq (v3.2) [3] with 'star_salmon' option.

Downstream analysis are using R (v4.2.1). Differential gene expression (DGE), Gene Ontology and deconvolution analysis were performed with DESeq2 (v1.38.3), clusterProfiler (v4.6.2) and DeconRNASeq (v1.40.0). Significant differential genes were defined with the padj <= 0.05(Benjamini-Hochberg) and |log2FoldChange| >= 0.6.

All scripts, with details of software versions, expression raw count files and results are freely available from https://github.com/CAD-ZM-BFX/Takaoka_Mallat_Goodall).

1. Patel, S., Haider, A., Alvarez-Guaita, A., Bidault, G., El-Sayed Moustafa, J.S., Guiu-Jurado, E., Tadross, J.A., Warner, J., Harrison, J., Virtue, S., et al. (2022). Combined genetic deletion of GDF15 and FGF21 has modest effects on body weight, hepatic steatosis and insulin resistance in high fat fed mice. Mol Metab *65*, 101589. 10.1016/j.molmet.2022.101589.
2. Di Tommaso, P., Chatzou, M., Floden, E.W., Barja, P.P., Palumbo, E., and Notredame, C. (2017). Nextflow enables reproducible computational workflows. Nat Biotechnol *35*, 316-319. 10.1038/nbt.3820.
3. Ewels, P., Magnusson, M., Lundin, S., and Kaller, M. (2016). MultiQC: summarize analysis results for multiple tools and samples in a single report. Bioinformatics *32*, 3047-3048. 10.1093/bioinformatics/btw354.

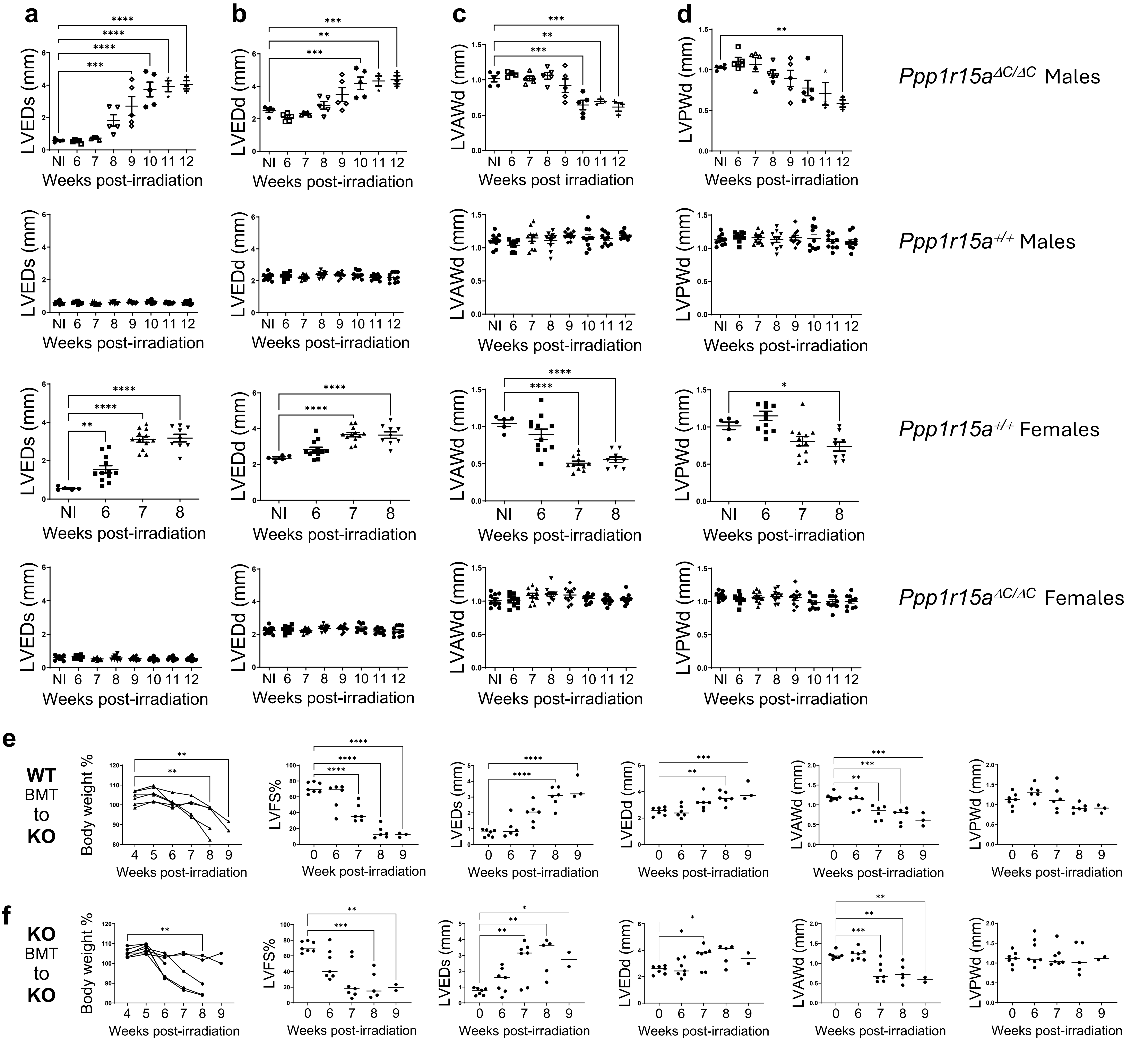

Figure S1. Related to Figure 1. Mice lacking functional PPP1R15A exhibit changes in heart function and geometry following whole body irradiation.

Ppp1r15a$^{+/+}$ or *Ppp1r15a*$^{\Delta C/\Delta C}$, male and female mice were irradiated (11Gy) followed by BM transfer from *Ppp1r15a*$^{+/+}$ mice. Echocardiography-derived parameters over time were compared with parameters from non-irradiated male mice (NI). Echocardiography of the parasternal short axis of the heart was used to assess (**a**) LV end diameter at systole, LVEDs, (**b**) LV end diameter at diastole LVEDd, (**c**) LV anterior wall thickness, LVAWd, and (**d**) LV posterior wall diameter, LVPWd. To determine the effect of bone marrow genotype on the development of irradiation-induced heart failure, irradiated *Ppp1r15a*$^{\Delta C/\Delta C}$ mice were reconstituted with bone marrow from either **(e)** *Ppp1r15a*$^{+/+}$ (WT) or **(f)** *Ppp1r15a*$^{\Delta C/\Delta C}$ (KO) mice, and monitored for body weight, shown as % of body weight compared to NI. Echocardiography of parasternal short axis to assess left ventricular fractional shortening (LVFS%) and LVEDs, LVEDd, Law, and LVPWd. Statistical analysis using one-way ANOVA and Dunnett's post-comparison test. *p < 0.05 **p < 0.01, ***p < 0.001, ****p < 0.0001.

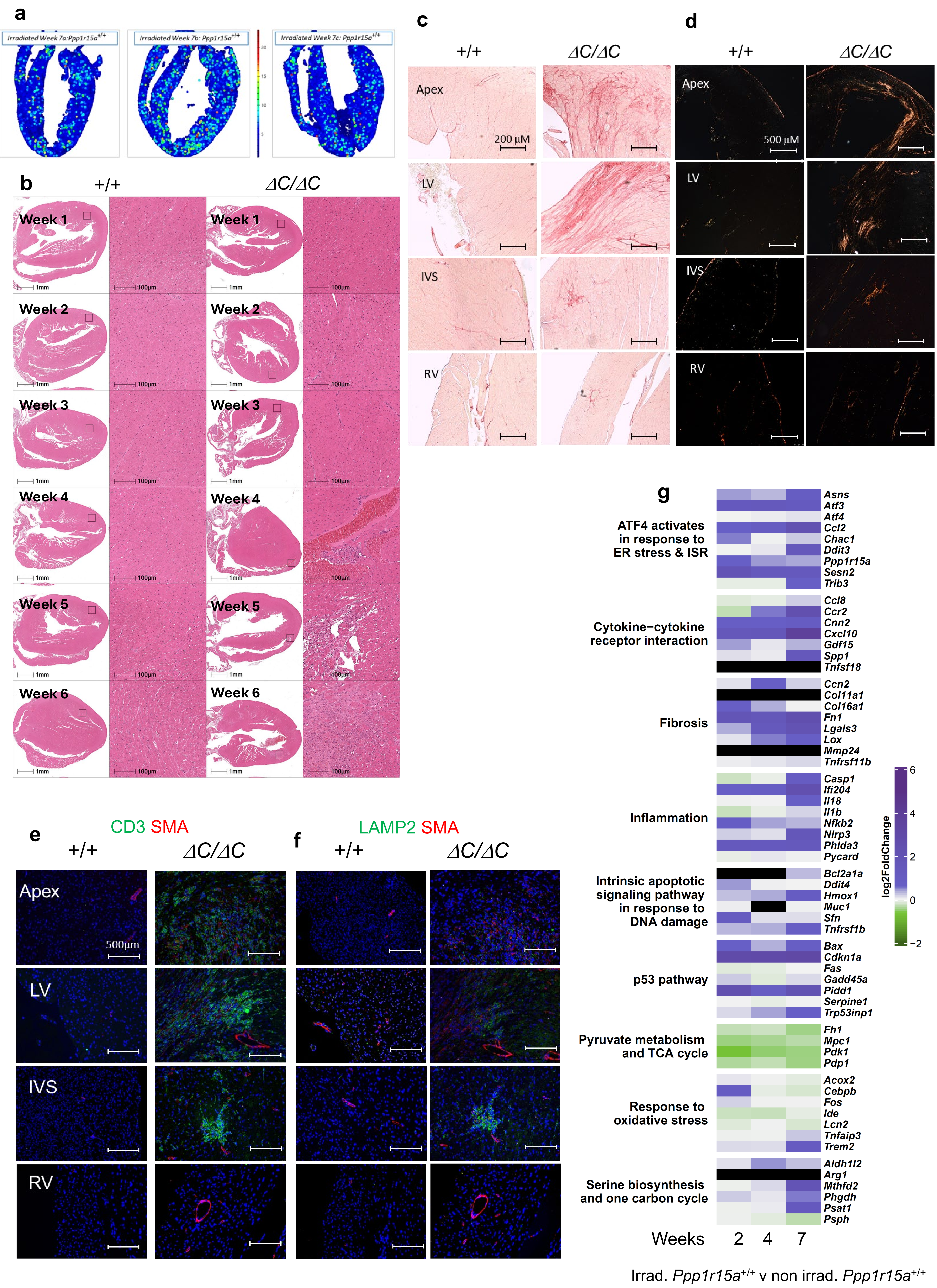

a
Irradiated Week 7a: Ppp1r15a+/+
Irradiated Week 7b: Ppp1r15a+/+
Irradiated Week 7c: Ppp1r15a+/+
b
+/+
ΔC/ΔC
Week 1
Week 2
Week 3
Week 4
Week 5
Week 6
1mm
100µm
c
+/+
ΔC/ΔC
Apex
LV
IVS
RV
200 µM
d
+/+
ΔC/ΔC
Apex
LV
IVS
RV
500 µM
e
CD3 SMA
+/+
ΔC/ΔC
Apex
LV
IVS
RV
500µm
f
LAMP2 SMA
+/+
ΔC/ΔC
g
ATF4 activates in response to ER stress & ISR
Asns
Atf3
Atf4
Ccl2
Chac1
Ddit3
Ppp1r15a
Sesn2
Trib3
Cytokine−cytokine receptor interaction
Ccl8
Ccr2
Cnn2
Cxcl10
Gdf15
Spp1
Tnfsf18
Fibrosis
Ccn2
Col11a1
Col16a1
Fn1
Lgals3
Lox
Mmp24
Tnfrsf11b
Inflammation
Casp1
Ifi204
Il18
Il1b
Nfkb2
Nlrp3
Phlda3
Pycard
Intrinsic apoptotic signaling pathway in response to DNA damage
Bcl2a1a
Ddit4
Hmox1
Muc1
Sfn
Tnfrsf1b
p53 pathway
Bax
Cdkn1a
Fas
Gadd45a
Pidd1
Serpine1
Trp53inp1
Pyruvate metabolism and TCA cycle
Fh1
Mpc1
Pdk1
Pdp1
Response to oxidative stress
Acox2
Cebpb
Fos
Ide
Lcn2
Tnfaip3
Trem2
Serine biosynthesis and one carbon cycle
Aldh1l2
Arg1
Mthfd2
Phgdh
Psat1
Psph
Weeks 2 4 7
log2FoldChange
6
4
2
0
−2
Irrad. Ppp1r15a+/+ v non irrad. Ppp1r15a+/+

Figure S2. Related to Figure 2. Irradiated *Ppp1r15a*$^{+/+}$ and *Ppp1r15a*$^{\Delta C/\Delta C}$ mice exhibit histological differences in heart tissue. (**a**) Spatial plots of *Ppp1r15a* expression assessed by Single Molecule In Situ Hybridisation (SM-ISH) in heart tissue sections derived from irradiated female WT mice, at 7 weeks post-irradiation. (**b**) Comparison of single *Ppp1r15a*$^{\Delta C/\Delta C}$ or *Ppp1r15a*$^{+/+}$mice hearts from female mice, 1-6 weeks post-irradiation stained using H&E stain.

Comparison of identical hearts shown in Figure 1a, 7 weeks post-irradiation, at 4 different locations – the apex, left ventricle (LV), interventricular septum (IVS), and right ventricle (RV). Sirius Red stain under (**c**) brightfield and (**d**) polarized light. Immunofluorescence staining for (**e**) CD3 (green) and (**f**) LAMP2 (Green), with smooth muscle actin (SMA) (red). The images were captured at equivalent locations and are representative of three hearts of each genotype. (**g**) Heatmap of top DEGs related to the selected gene ontology pathways. Log2Fold changes are shown for weeks 2, 4,7 post-irradiation *of Ppp1r15a*$^{+/+}$ mice relative to the non-irradiated *Ppp1r15a*$^{+/+}$ control. The Black cell colour represents genes not identified in the corresponding DEGs analysis.

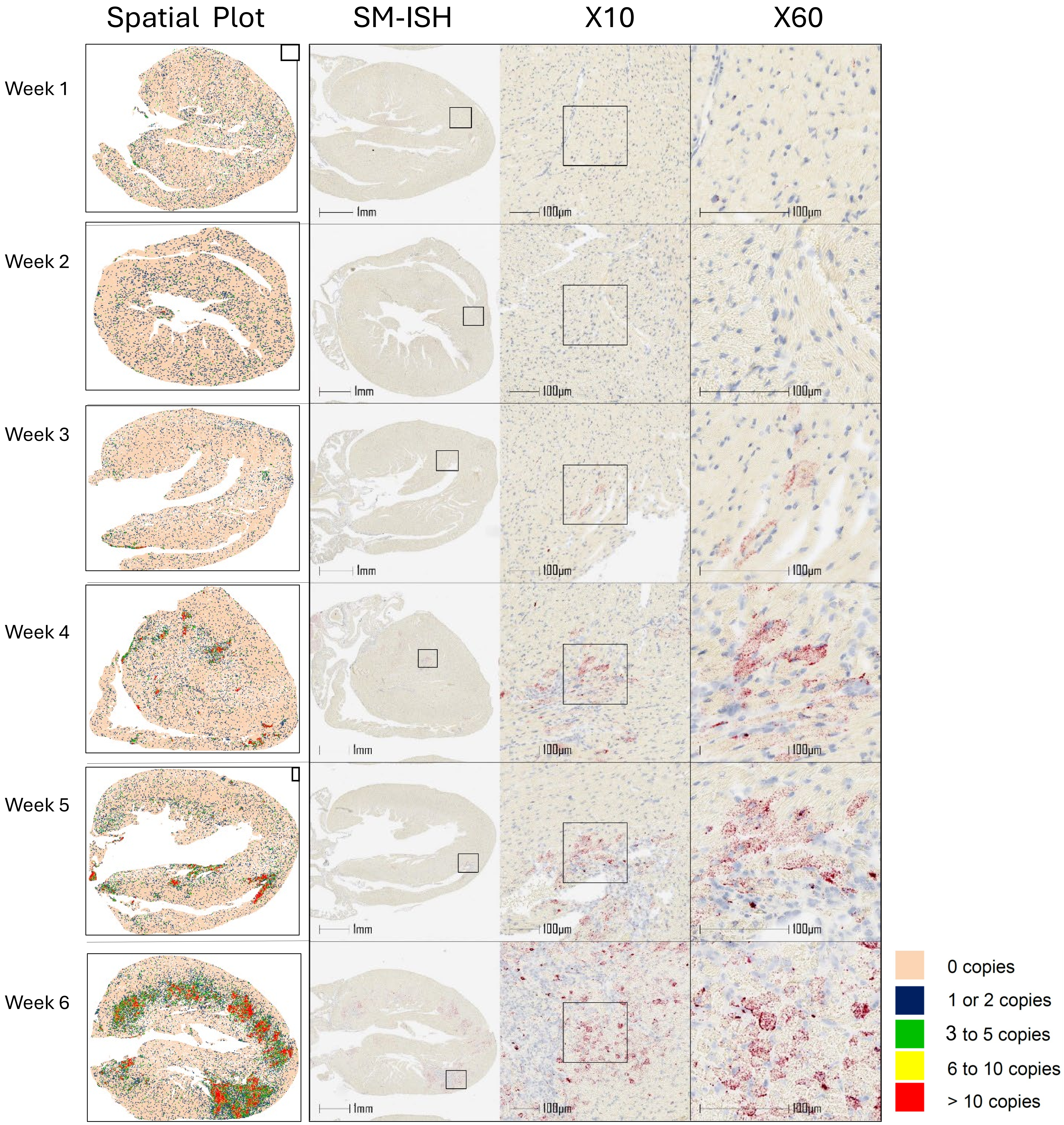

Figure S3. Related to Figure 3. Progression of *Gdf15* transcript expression in heart tissue following whole body irradiation of *Ppp1r15a*$^{\Delta C/\Delta C}$ Mice.

Ppp1r15a$^{\Delta C/\Delta C}$ female mice (littermates) were irradiated (11Gy), followed by BM transfer and culled at the time points indicated, heart tissue was analysed by Small Molecule In Situ Hybridisation (SM-ISH) for *Gdf15* (red spots). Spatial plot shows distribution of quantified *Gdf15* mRNA transcripts, categorised as 0 copies, 1 to 2 copies, 3 to 5 copies, 6 to 10 copies and 10+ copies (see colour legend).

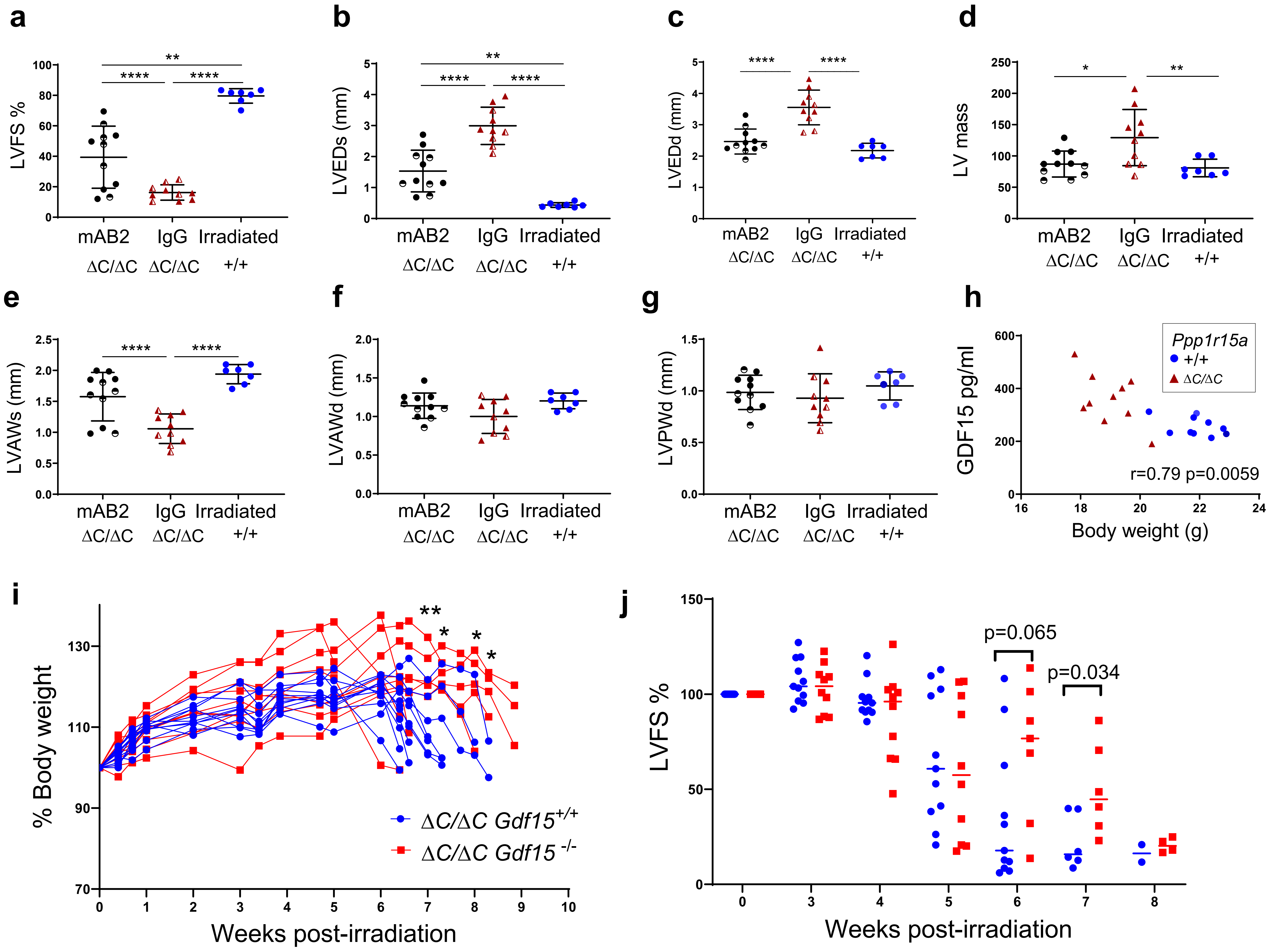

Figure S4. Related to figure 5. The GDF15 neutralising antibody, mAB2 changes parameters of heart function in irradiated mice lacking functional PPP1R15A. *Ppp1r15a*$^{\Delta C/\Delta C}$ ($^{\Delta C/\Delta C}$) or *Ppp1r15a*$^{+/+}$ (+/+) mice were irradiated (11Gy) and reconstituted with *Ppp1r15a*$^{+/+}$ bone marrow. At 4 weeks *Ppp1r15a*$^{\Delta C/\Delta C}$ mice were given antibody that blocks GDF15 activity (mAB2) or an isotype control antibody (IgG) as described in Figure 1. Left ventricular heart function was assessed by echocardiography and expressed as (**a**) LVFS %, (**b**) LVEDs, (c) LVEDd, (**d**) LV mass, (e) LVAWs, (**f**) LVAWd and (**g**) LVPWd. Female *Ppp1r15a*$^{\Delta C/\Delta C}$ mice are indicated using half-filled symbols. Data analysed using one way ANOVA and Tukey test post comparison analysis. (**h**) Correlation of plasma GDF15 with % body weight at 9 weeks post irradiation, r calculated by Pearson's correlation coefficient. *Ppp1r15a*$^{\Delta C/\Delta C}$ *Gdf15*$^{-/-}$ (n=11) or *Ppp1r15a*$^{\Delta C/\Delta C}$ *Gdf15*$^{+/+}$ (n=10) were irradiated (11Gy) and reconstituted with *Ppp1r15a*$^{-/-}$ bone marrow, and monitored over a time course (weeks 1-10) by (**i**) body weight and (**j**) echocardiography for left ventricular fractional shortening (LVFS%). Data analysed using t test on each time point. All data show mean +/- SD. *p < 0.05 **p < 0.01, ***p < 0.001, ****p < 0.0001 or exact P value.

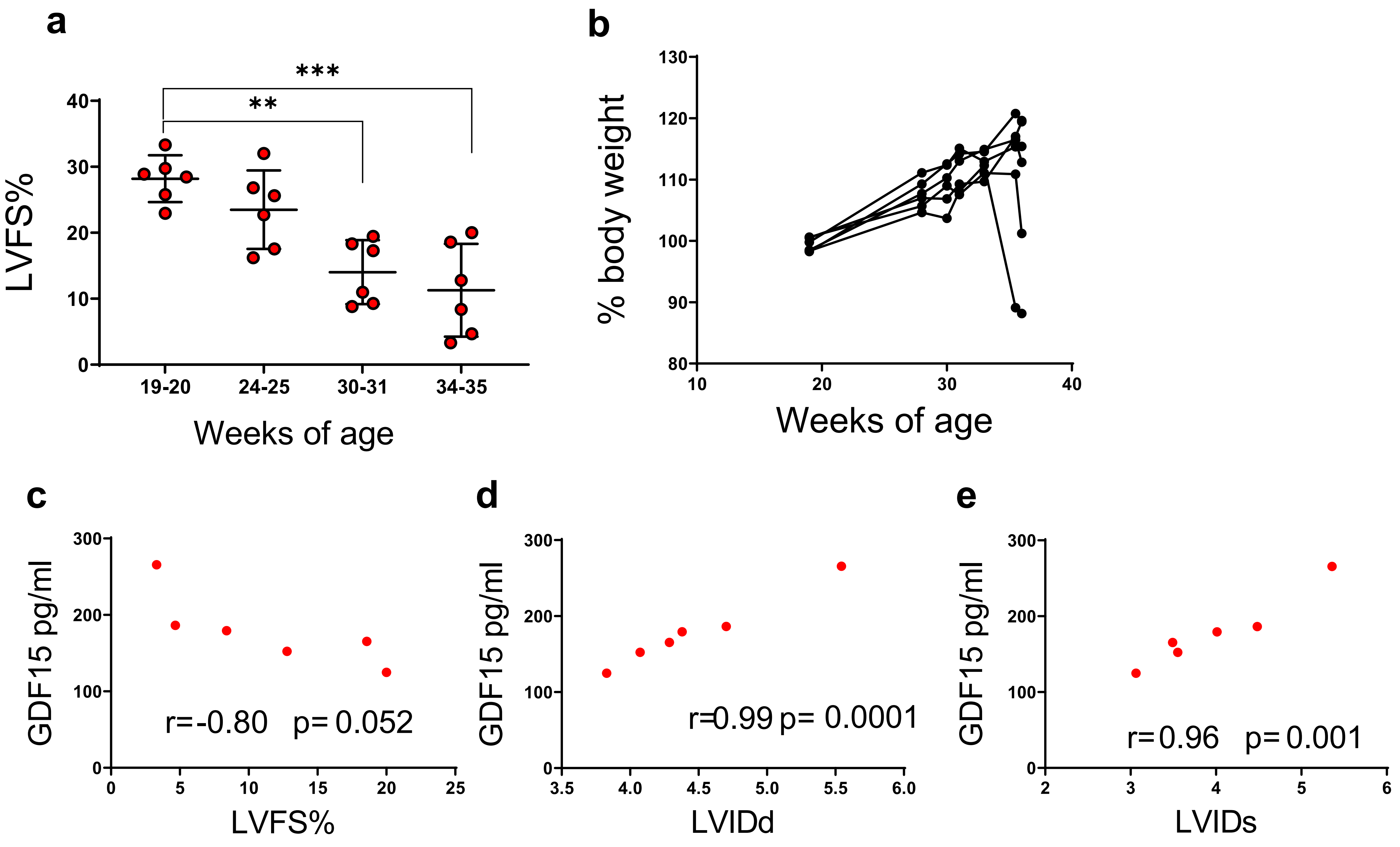

Figure S5. GDF15 expression correlates with parameters of heart function and geometry in cYKO mice that have a cardiomyocyte specific deletion of Yme1l. cYKO mice that lack Yme1l in cardiomyocytes exhibit (**a**) reduced heart function at 34-35 weeks of age. (**b)** a proportion of the mice also exhibit dramatic weight loss. Plasma GDF15 collected at 34-5 weeks of age, correlates with changes in parameters of heart geometry and heart function (**c-e**). r calculated by Pearson's correlation coefficient.
